# Extracellular Vesicles in Rheumatoid Arthritis and Systemic Lupus Erythematosus: Functions and Applications

**DOI:** 10.3389/fimmu.2020.575712

**Published:** 2021-01-14

**Authors:** Bo Zhang, Ming Zhao, Qianjin Lu

**Affiliations:** ^1^ Department of Dermatology, Second Xiangya Hospital, Central South University, Hunan Key Laboratory of Medical Epigenomics, Changsha, China; ^2^ Clinical Immunology Research Center, Central South University, Changsha, China; ^3^ Research Unit of Key Technologies of Diagnosis and Treatment for Immune-related Skin Diseases, Chinese Academy of Medical Sciences (2019RU027), Changsha, China

**Keywords:** extracellular vesicles, exosome, systemic lupus erythematosus, microRNA, rheumatoid arthritis

## Abstract

In the last two decades, extracellular vesicles (EVs) have aroused wide interest among researchers in basic and clinical research. EVs, small membrane vesicles are released by almost all kinds of cells into the extracellular environment. According to many recent studies, EVs participate in immunomodulation and play an important role in the pathogenesis of autoimmune diseases. In addition, EVs have great potential in the diagnosis and therapy of autoimmune diseases. Here, we reviewed the latest research advances on the functions and mechanisms of EVs and their roles in the pathogenesis, diagnosis, and treatment of rheumatoid arthritis and systemic lupus erythematosus.

## Introduction

Rheumatoid arthritis (RA) and systemic lupus erythematosus (SLE) are both autoimmune diseases that can involve multiple organs. Their etiologies and pathogenesis are complex, and epigenetic and environmental factors are shown to be associated with the onset of the disease (1, 2). Glucocorticoids, immunosuppressants, and biological agents are commonly used in the treatment of RA and SLE, but problems such as toxic side effects and non-response to treatment remain (3–5). EVs are phospholipid bilayer-enclosed vesicles secreted from all cell types. The classification of EVs includes exosomes (<150 nm), microvesicles (150–1,000 nm) (6), and apoptotic bodies (1,000–5,000 nm), depending on size and biogenesis (7). EVs play an important role in cellular communication processes. In the past, intercellular communication was thought to have two modes, direct contact between cells and secretion of cellular molecules (8). The relationship between EVs and cellular communication has attracted more attention and has become the third mechanism of intercellular communication (9). EVs began to be isolated and studied from additional cell types, such as immune cells, nerve cells and tumor cells (10). It is demonstrated that EVs are involved as carriers in intercellular communication by transporting lipids, proteins, and other components (11). In 1996, Raposo et al. first showed that EVs could stimulate adaptive immune responses (12). EVs can also carry mitochondria to regulate immunity and alter the phenotype of macrophages (13).

EVs are secreted by almost every functional cell type and have the characteristics of easy detection and stability. Body fluids, such as urine and peripheral blood contain EVs, which present promising prospects as biomarkers for tumors, infectious diseases, and autoimmune diseases. Furthermore, the biological characteristics of EVs that can transport multiple cellular components also make it possible to use them in therapeutic approaches for diseases. To date, there have been some studies of EV treatment for RA and SLE, which have made certain achievements (14, 15). Here, we summarize the functions of EVs on immune cells and their applications in the pathogenesis, diagnosis and treatment of RA and SLE.

## The Biogenesis and Composition of EVs

Different types of EVs have slightly different biological origins, and their biological functions are determined by their respective intercellular components. Exosomes are EVs with a diameter of no more than 150 nm. The limiting membrane of late endosomes generates exosomes by invagination and budding (16). Then, exosomes are covered by endosomal multivesicular bodies (MVBs) and form intraluminal vesicles (ILVs), which fuse with the plasma membrane and are exocrine. The endosomal sorting complex required for transport (ESCRT) is also involved in exosome generation, which is formed by approximately twenty proteins (17). The action of ESCRT is mainly carried out by four proteins following specific steps. First, ESCRT-0 recognizes ubiquitinated proteins in the endosomal membrane and isolates them individually. Second, both ESCRT-I and ESCRT-II mediate the transformation and assembly of the membrane. Third, ESCRT-III leads to the scission (18). Exosomes contain proteins, nucleic acids, lipids, and organelles such as mitochondria (19).

Microvesicles (MVs) are formed as the plasma membrane germinates outward directly. Although the diameter-based classification of exosomes and MVs is somewhat controversial, the fundamental distinction is apparent based on their biogenesis. Their formation is related to changes in the symmetry of phospholipids in cell membranes, and their release is associated with lipid rafts on the cell membrane (20). Proteins and phospholipids are unevenly distributed on the plasma membrane by the regulation of aminophospholipid translocases. The transfer of phosphatidylserine and the change in protein structure create a dynamic equilibrium, contributing to the formation of MVs (21). MVs are composed similarly to exosomes.

Apoptotic bodies are the products of apoptosis, while exosomes and MVs are secreted by living cells. The contents of the cell after apoptosis decompose into membrane-bound vesicles. In terms of composition, apoptotic bodies are characterized by the inclusion of organelles and smaller vesicles (21). Apoptotic bodies also contain ribosomal RNA which are almost undetectable in exosomes and MVs (22). They work primarily as garbage carriers of cells containing cellular wastes (23).

## The Role and Mechanism of EVs in The Immune System

Research on EVs began in 1983 when exosomes were first identified in reticulocytes from sheep (24). However, it was not until 1996 that B cells were shown to release exosomes with the major histocompatibility complex class II (MHC II), which indicated the relationship between immune cell regulation and exosomes (12). Other immunocytes, such as T cells, natural killer (NK) cells, and dendritic cells (DCs), have been proven to be associated with EVs in recent publications (25–27). Since EVs, especially exosomes, can carry MHC II, it is possible for EVs to participate in antigen presentation. Tian et al. summarized three mechanisms by which EVs are involved in antigen presentation (7). First, loading antigen proteins inside exosomes improves the efficiency of antigen presentation, and then APCs costimulating molecules act on the activation of T cells (28). Second, when peptide/MHC complexes are formed, exosomes with antigens can be captured by APCs and then are exposed to the cell membrane to activate T cells. Third, EVs directly activate T cells without the participation of APCs (29). Interestingly, reverse transport of miRNA by exosomes, which is antigen-driven, has been proven to regulate the gene expression of APCs (30).

DCs are one of the most effective immunocytes in presenting antigens and are critical to both innate and adaptive immunity. Some DCs can establish immune tolerance by reducing the T cell activity level, while the other DCs can activate T cells to enhance the immune response. With the expression of a high level of MHC I/peptide complexes as well as B7 and ICAM-1, exosomes from DCs are able to directly activate CD8^+^ T cells without the participation of normal APCs (31). Not only the mutual effect between DCs and T cells but also the intercellular communication between DCs play crucial roles in the process of DCs regulating innate immune responses (32). Angela Montecalvo et al. found that the miRNA components of exosomes released by DCs at different stages of maturation were different. Mature DC-derived exosomes show a stronger T-cell stimulatory ability than immature DC-derived exosomes because of higher expression of CD86 and CD54 (32). DC-derived exosomes (Dexs) containing MHC/peptide complexes can boost T cell-dependent tumor rejection. And NK cells can be activated by both IL-15Ra and NKG2D ligands in Dexs and secrete IFN-*γ* (33).

Similar to other APCs, B cells have a cellular structure called the endocytic compartment MIIC (major histocompatibility complex [MHC] class II-enriched compartment), which participates in the activation of antigen-specific MHC II-restricted T cell responses (12). Activated B cells infected with EBV can also excrete exosomes with EBV-miRNAs, which accumulate in neighboring primary immature monocyte-derived DCs (MoDCs) without infection (34). Furthermore, exosomes from activated B cells with EBV infection harbor the viral latent membrane protein 1 (LMP1), which imitates CD40 signaling, resulting in the propagation of B cells as well as T cell-independent class-switch recombination (35).

**Figure 1:**
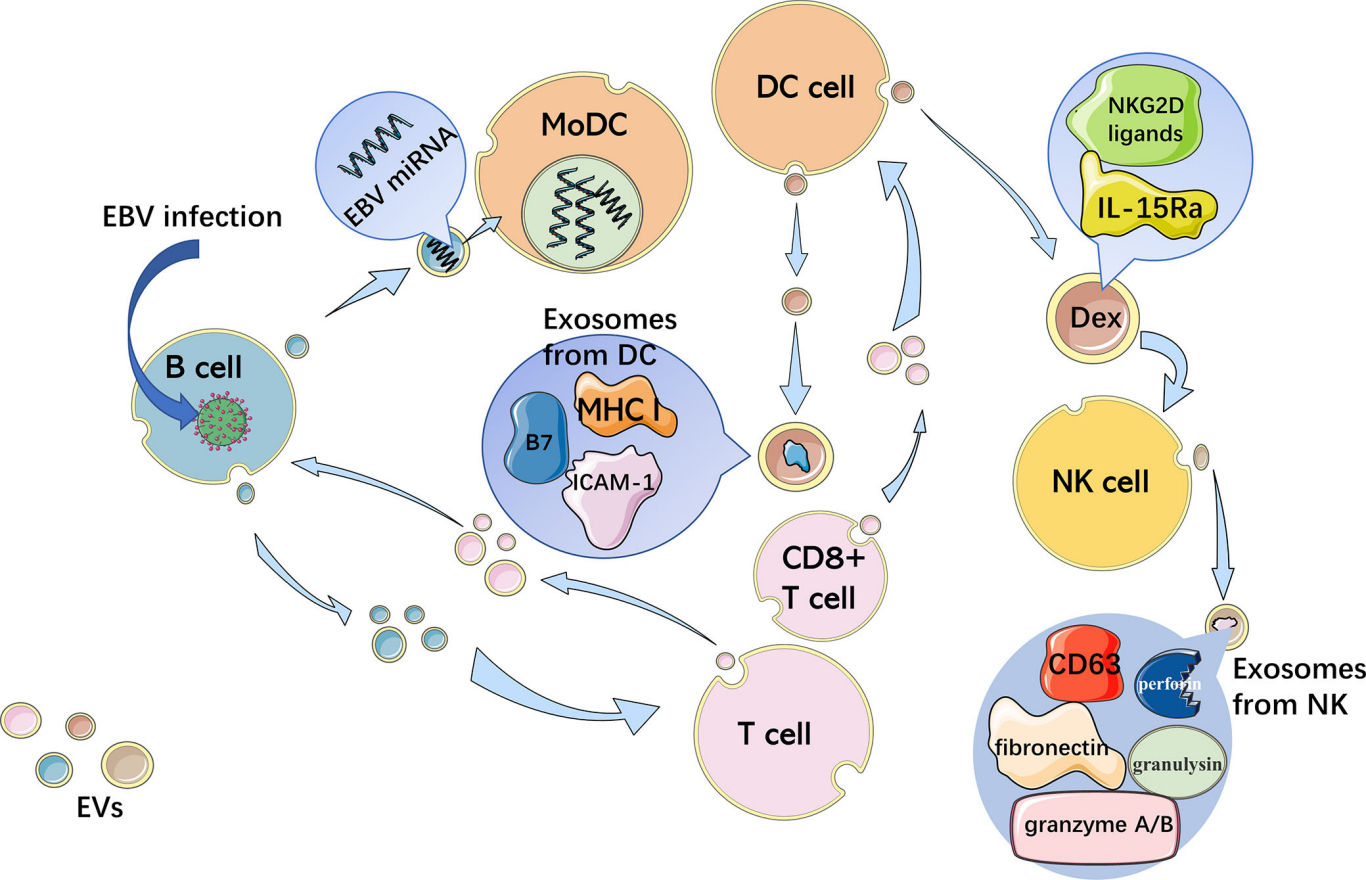
Interaction between immune cells through EVs. APCs activate T cells with EVs and reverse transport of miRNA by exosomes, which is antigen-driven, regulating the gene expression of APCs. B cells infected with EBV excrete exosomes with EBV-miRNAs, accumulating in neighboring MoDCs. Exosomes with MHC I/peptide complexes, B7 and ICAM-1 from DCs, directly activate CD8+ T cells. Dexs containing IL-15Ra and NKG2D ligands activate NK cells, which then secrete exosomes with CD63, fibronectin, perforin, granulysin, and granzyme A/B.

NK cells are important immunocytes in innate immunity with a variety of biological functions, including recognizing and killing viral infections and tumor cells, and producing cytokines such as interferon (IFN)-*γ*, involved in immune regulation (36). After being activated by Dexs, NK cells secrete exosomes containing CD63, fibronectin, perforin, granulysin, and granzyme A/B, which indicates that NK-derived EVs contain the killing function of NK cells (26). The EV interaction between APCs and T cells as well as NK cells is shown in **Figure 1**.

Other non-immune cells can also produce EVs and participate in the regulation of immune responses. EB virus (EBV) infected cells can secrete exosomes containing EBV-microRNAs (miRNA) to mediate gene silencing in immune cells (37). EVs derived from tumor cells and stem cells have also been shown to regulate immune function (38). miRNAs in tumor exosomes may induce immune tolerance (39). While EVs derived from stem cells have been shown to regulate immunity and reduce inflammatory responses (40, 41). Lipid-filled vesicles, derived by adipocytes, can modulate tissue macrophage to participate in immune regulation (42).

## The Role of EVs in RA

### EVs Are Involved in the Pathogenesis of RA

RA is an autoimmune disease with a high incidence that damages multiple joints throughout the body and can cause progressive disability. RA is characterized by synovial inflammation and cartilage destruction (43). In comparison with those from normal controls, EVs showed a high density in the synovial fluid of RA samples, which was associated with disease progression (44). EVs are mainly involved in antigen presentation, inflammatory cytokine and miRNA transmission, and activation of fibroblast-like synoviocytes (FLSs) in the pathological process of RA. It has also been shown that FLSs-derived EVs contained hexosaminidase D activities in the samples of RA patients (45). Additionally, the level of N-acetyl-beta-D-hexosaminidase (NAHase) in destructive RA is higher than that in inflammatory RA, indicating that glycosaminoglycan-degrading glycosidases may cause joint damage in RA (46). Citrullinated proteins can be detected in synovial exosomes, which can enhance T cell activity with fibronectin (47). In addition, antibodies to citrullinated protein antigens (ACPAs) are crucial in the pathological process of RA and are expected to work as biomarkers with the highest predictive value (48, 49).

FLS-derived microparticles (MPs) contain B cell stimulation factors in the synovial fluid of the joints of RA patients (50). There are microparticle-containing immune complexes (mpICs) in synovial fluid with CD41 highly expression, indicating platelet-derived. These mpICs could induce neutrophils to release leukotrienes, which proves that platelet mpICs are proinflammatory and highly reactive (51). Also, platelet-derived microparticles (MPs) seem to release IL-1β, which promotes joint inflammation by increasing the levels of IL-6 and IL-8 in fibroblasts from RA patients (52). Tumor necrosis factor-α (TNF-α) is crucial to the pathogenesis of RA. TNF-α binding membranes were detected in FLSs-derived EVs from RA patients. EVs containing TNF-α activated AKT and NF-*κ*B and rendered these activated T cells resistant to apoptosis (53). Additionally, T cell-derived MPs treated with TNF-α could upregulate prostaglandin E2 (PGE2), microsomal prostaglandin E synthase 1 (mPGES-1) and cyclooxygenase 2 (COX-2) (54). Then COX2 caused pain and inflammation in patients. Coinhibitory T cell receptors can be expressed in cells from RA joints, including PD-1 and TIM-3. EVs from synovial fluid and T cells after cocultivation could express PD-1. Carrying the PD-1 receptor and inhibitive miRNAs, EVs may induce T cell exhaustion (55).

The transmission of miRNAs is crucial in the RA pathological process. Of all the miRNAs associated with RA, miR-155 and miR-146a have attracted most attention. A study proved that exosomal miR-146a and miR-155 are expressed at high levels in RA synovial tissue (56). Furthermore, miR-155 can be upregulated by stimulation with proinflammatory mediators, including Toll-like receptor (TLR) ligands, TNFα and IL-1β. Overexpression of miR-155 in RA synovial fibroblasts (RASFs) can downregulate matrix metalloproteinase 3 (MMP-3) and MMP-1 (57). MMP-3 is involved in the generation of severe cartilage damage (58). All these components in EVs contribute to the onset and development of RA (**Table 1**).

**Table 1:** EVs involved in the pathogenesis of RA and SLE.

Disease	Molecular/Parameter	Reference
RA	Density of EVs	(44)
	FLSs-derived EVs contained hexosaminidase D	(45)
	Citrullinated proteins in exosomes	(47)
	Microparticles-containing immune complexes	(51)
	Platelet-derived Microparticles	(52)
	TNF-α contained in EVs	(53)
	Exosomal miR-155	(57)
	Exosomal miR-146a	(8)
SLE	Exosomal miR-146a	(59)
	Exosomal miR-21	(60)
	Exosomal miR-574	(61)
	MVs from apoptosis	(62)

### The Role of EVs in Diagnosis and Treatment of RA

Existing studies implied that EVs have potential as biomarkers for RA. RA patients with IgM-rheumatoid factor (RF) EVs showed high-level C-reactive protein (CRP) and Erythrocyte sedimentation rate (ESR) levels compared with those of RA patients without IgM-RF in EVs (63). Thus, EVs with IgM-RF can be used to distinguish between active and inactive RA. RA patients express high levels of MPs in the circulatory system compared with those in healthy controls (64). Moreover, as the role of miRNA in the pathology of RA disease has been revealed, exosomal miRNAs, including miR-155 and miR-146a, can be used for the early diagnosis of RA (57). Potential biomarkers for RA in EVs are summarized in **Table 2**.

**Table 2:** EVs as potential biomarkers for RA and SLE.

Disease	Molecular	Change	Body fluids	Reference
RA	IgMRF^+^ EVs	Positive correlation with disease activity	Serum	(65)
	CD41^+^EVs	Upregulated	Synovial fluid	(44)
	CD3^+^CD8^+^Tcell-derived EVs	Upregulated	Synovial fluid	(66)
	Hotair in EVs	Upregulated	Serum/urine	(67)
	Exosomal miR-106b	Upregulated	Synovial fluid	(68)
	Exosomal miR-6089	Downregulated	Serum	(69)
	Exosomal miR-548a-3p	Downregulated	Serum	(70)
	Citrullinated proteins in EVs	Upregulated	Synovial fluid	(47)
	miR-146a and miR-155	Upregulated	Synovial fluid /plasma	(71)
SLE	Urinary exosomal miR-135b-5p, miR-107, miR-31	Upregulated in LN	Urine	(72)
	Urinary exosomal miR-21, miR-150, and miR-29c	Correlated with LN chronicity index (CI)	Urine	(73)
	Exosomal miR-146a	Downregulated	Serum	(74)
	Urinary podocyte-derived MPs	Positively correlated with the SLE Disease Activity Index (SLEDAI) score	Urine	(75)
	Endothelial cells-MPs	Upregulated	Serum	(76)
	Urinary MP-HMGB1	Upregulated in active LN than inactive	Urine	(77)
	Monocytic CD 14^+^ MP	Positively correlated with the disease activity in SLE	Plasma	(65)

Mesenchymal stem cells (MSCs) have anti-fibrosis and anti-inflammatory immune regulatory effects. The transplantation of MSCs has been used as a new technique for RA therapy. When collagen-induced arthritis (CIA) rats were treated with human umbilical cord MSCs (hUCMSCs), the results showed that hUCMSCs can reduce T lymphocyte activity and function, as well as inhibit Th17 cells and induce Treg cells to alleviate the disease (78). The same immunomodulatory function between hUCMSCs and hUCMSC-derived EVs has been demonstrated *in vitro*, which indicates the potential of hUCMSC-derived EVs as a new treatment for RA (78). hUCMSC-derived EVs also can inhibit the expression of IL-17 by downregulating Th17 cells and increasing the proportion of Treg cells in a dose-dependent manner. Moreover, it was demonstrated that periarticular injection of exosomes containing IL-10 or exosomes from bone marrow-derived DCs could relieve arthritis by anti-inflammatory action since DC-derived exosomes showed strong anti-inflammatory and immunosuppressive activity through the class II-dependent pathway. In addition, as exosomes are phenotypically stable after purification *in vitro* (79), EVs could potentially be a drug carrier for precise therapy for RA. Louise et al. used the human neutrophil-derived EVs as scaffolds, which have the function of immune regulation and cartilage penetration. Anti-reactive oxygen species-collagen type II (Anti-ROS-CII) is an antibody targeting impaired arthritic cartilage. Combining this antibody with EVs allows the complex to penetrate the cartilage into the articular cavity and still maintain antibody activity, suggesting the potential of EVs as a targeted carrier for drug delivery (80).

## The Role of EVs in SLE

### EVs Are Involved in the Pathogenesis of SLE

SLE is a complex heterogenous autoimmune disease that involves damage to multiple organs throughout the body and can cause death in severe cases. Patients with SLE are characterized by T and B lymphocyte dysfunction, accumulation of autoantibodies, and deposition of immune complexes (81). However, the pathogenesis of SLE remains unclear. The role of EVs in the pathogenesis of SLE is of interest to researchers.

Exosomal miRNAs in exosomes play an important role in the development of SLE. The level of miR-146a contained within exosomes in the urine of lupus patients was significantly higher than that outside of exosomes. In contrast, miR-146a levels in serum exosomes were significantly lower in SLE patients than in HCs (59). Of all miRNAs, miR-146a can significantly distinguish active LN from inactive LN and is related to inflammation and fibrosis of the kidney (74). In addition, miR-146a may be upregulated by chemokines as well as proinflammatory cytokines and leads to anemia in SLE patients (82). MSCs can internalize exosomes with miR-146a and target TRAF6/NF-*κ*B signaling, leading to the senescence of MSCs (59). The senescence of MSCs may be related to the disease activity and pathological process of SLE (83, 84). Another important exosomal miRNA is miR-21 contained in EVs, facilitating estrogen-regulated STAT1 activation and Toll-like receptor (TLR) 8 expression in SLE. miRNAs can be endogenous ligands of human TLR7, which is the single-stranded RNA (ssRNA) receptor expressed by plasmacytoid dendritic cells (pDCs). miR-21 can replace viral ssRNA to combine with TLR8 to stimulate innate immune responses (60). Interferon (IFN)-α plays a major role in SLE (85). It was proven that miRNAs in exosomes, such as miR-574, upregulated type I IFNs secreted by pDCs in SLE (61). MVs from apoptosis in SLE serum can activate cyclic guanosine monophosphate (GMP)-AMP synthase (cGAS), which stimulates the stimulator of interferon genes (STING) pathway and upregulates the type I IFN production (62). The EVs involved in the pathogenesis of SLE were shown in **Table 1**.

### The Role of EVs in Diagnosis and Treatment of SLE

EVs can be used to measure disease activity and differential diagnosis in patients with LE. Damage to glomerular podocytes is crucial in renal injury in SLE. Urinary podocyte-derived MPs can be used for the prediction of disease activity. They are positively correlated with clinical indicators of SLE, including erythrocyte sedimentation rate, proteinuria, and SLE Disease Activity Index (SLEDAI) score (75). Urinary HMGB1 in MPs is expressed at a significantly high level in active LN, which can distinguish between active and non-active LN (77). And identification of MPs with different surface proteins in SLE patients can predict disease activity and vascular damage (86). It was reported in another study that high plasma expression of monocytic CD 14^+^ MP has a positive correlation with the disease activity of SLE (65). Compared with healthy controls and systemic sclerosis (SSc) patients, SLE patients presented a higher expression of endothelial cell MP (EMP), suggesting that EMP has potential as a biomarker for SLE vascular lesions (76). Potential biomarkers for SLE in EVs are summarized in **Table 2**.

EVs have also received further attention in the treatment of SLE. In LN, MP surface proteins, especially G3BP, play a key role in the deposition of ICs. Therefore, targeting MPs may be a new approach for treating LN (87). MSC-derived MVs have anti-inflammatory and immunomodulatory effects (88). Although the use of MSCs in the treatment of SLE is mature and has been used clinically (89–93), Juhi et al. found that MSC-derived EVs can replace MSCs in the treatment of SLE, with the following advantages. First, there is no evidence that EVs are carcinogenic. Second, compared with MSCs, EVs are more stable and easier to preserve in the long term. Third, EVs do not cause an immune response that harms the host. EVs can bypass the blood–brain barrier, which makes it possible for EVs to be used in the treatment of lupus encephalopathy (94). In addition, EVs are easier to prepare on a large scale and at a low cost for clinical therapy. However, the effect of EVs is closely related to the dose, and the appropriate therapeutic dose needs to be explored.

## Conclusion and Outlook

Although EVs were discovered in 1983, research on EVs has grown rapidly only in the current century. The role of EVs in cellular communication and immune regulation is being gradually explained. EVs secreted by immune cells are involved in antigen presentation and regulation of immunity. Cytokines or miRNAs contained in EVs and MSC-derived EVs play important roles in autoimmune diseases. Technology for isolating and purifying EVs is growing (95–98). New technologies, such as nanoscale flow cytometry (NanoFCM) and microfluidic platforms with 100,000 pillars, have been used for more efficient isolation of EVs (99, 100). A microfluidic cell culture platform using a 3D-printed microfluidic chip has also been used in the preparation of EVs (101). The research development of EVs is helpful to understand the pathogenesis of autoimmune diseases and provide new ideas for diagnosis and treatment. At the same time, we should also pay attention to the role of EVs in the onset and development of diseases and emphasize the dose and safety in the treatment to avoid potential side effects.

## Author Contributions

BZ wrote the manuscript. MZ and QL conceptualized and revised the manuscript. All authors contributed to the article and approved the submitted version.

## Funding

The present research was supported by the National Natural Science Foundation of China (No. 81874243, No. 81861138016, No. 81830097), CAMS Innovation Fund for Medical Sciences (CIFMS) (2019-I2M-5-033), the Key project for international and regional cooperation in science and technology innovation of Hunan province (2019WK2081), and the Project for leading talents in science and technology in Hunan province (2019RS3003).

## Conflict of Interest

The authors declare that the research was conducted in the absence of any commercial or financial relationships that could be construed as a potential conflict of interest.
